# A Novel Prognostic Index Based on the Analysis of Glycolysis-Related Genes in Head and Neck Squamous Cell Carcinomas

**DOI:** 10.1155/2020/7353874

**Published:** 2020-09-24

**Authors:** Yuchao Liu, Shihua Yin

**Affiliations:** Department of Otolaryngology and Head and Neck Surgery, Second Affiliated Hospital of Guangxi Medical University, Nanning 530021, China

## Abstract

**Aims:**

The preferential dependence on glycolysis as a pathway of energy metabolism is a hallmark of cancer cells. However, the prognostic significance of glycolysis-related genes in head and neck squamous cell carcinoma (HNSCC) remains obscure. The purpose of this study was to identify glycolysis-related genes of prognostic value in HNSCC.

**Results:**

Transcriptional and clinical data of 544 HNSCC samples were obtained from The Cancer Genome Atlas (TCGA) dataset. By gene set enrichment analysis (GSEA) and by employing a univariate and subsequently a stepwise multivariate Cox proportional regression model, eight glycolysis-related genes of prognostic significance in HNSCC (*KIF2A*, *JMJD8*, *HMMR*, *STC2*, *HK1*, *EXT2*, *GPR8*, and *STC1*) were identified. The patients were clustered into two groups (high and low risk) based on the expression of these genes. High-risk patients had significantly a shorter overall survival than low-risk patients. Furthermore, a new prognostic indicator based on selected glycolysis-related genes was developed by multivariate Cox analysis that proved to be a better predictor of patient outcome compared to other clinical factors.

**Conclusion:**

Our findings provide new insights into the role of glycolysis in HNSCC. The identified genes predict the patient prognosis and might substantially contribute to the development of individualized treatments.

## 1. Introduction

Head and neck squamous cell carcinoma (HNSCC) is a fatal malignancy and one of the leading causes of cancer death worldwide [1]. In particular, over 830,000 people are diagnosed with head and neck cancer each year, and more than 430,000 die from the disease [2]. HNSCC accounts for 90% of all head and neck cancers [3]. Although various treatment options are available, such as surgery, radiotherapy, chemotherapy, immunotherapy, targeted therapy, and combination therapy [4], patient survival is still poor. The five-year survival time ranges between 40 and 50% [5]. Given the high lethality of the disease, better tools for prognosis may help improve HNSCC management.

To date, HNSCC prognosis still mainly relies on histopathologic examination and tumor staging. However, these approaches are unsuitable for reliable prediction of patient outcome. Previous studies have reported accurate and quantitative paradigms for prognosis prediction based on molecular markers or critical gene profiles that may help optimize therapeutic regimens. However, additional molecular biomarkers for individualized therapy are urgently needed. Compelling evidence has suggested that increased glycolysis is a hallmark of cancer cells [6]. Even under normal oxygen concentrations, the rate at which cancer cells metabolize glucose through glycolysis increases compared to normal cells [7, 8]. This metabolic change increases glucose uptake and lactate production, thereby affecting cell growth, proliferation, angiogenesis, and invasion [5, 9, 10]. Glycolysis is a complex and rigorous process, which is strictly and finely regulated by related genes.

Similar to most aggressive tumors, HNSCC exhibits a high rate of glycolysis to meet its metabolic demands [11, 12]. Consistently, molecular imaging studies using 18F-fluoro-2-deoxy-d-glucose positron emission tomography demonstrated increased glucose uptake and glycolysis in HNSCC [13–15]. Moreover, an increasing number of studies have demonstrated that, in HNSCC, the changes in glycolysis are associated with alterations in oncogenes, tumor suppressor genes, as well as with the overexpression of glycolytic enzymes and glucose transporters [16]. Several studies have also attempted to predict the relationship between patient survival and glycolysis. For example, Grimm et al. demonstrated that the overexpression of *TKTL1* is negatively correlated with the survival of patients with oral squamous cell carcinoma [17]. These findings substantiate the involvement of glycolysis in HNSCC and highlight the potential of glycolysis-related genes as prognostic markers in this disease. Notably, the association between single glycolysis-related genes and HNSCC progression has already been addressed. However, screening and identification of molecular markers that predict the prognosis of HNSCC by using a wide range of glycolysis-related gene expression profiles has not been studied. The objective of our study was to explore the latent applied value of glycolysis-related genes in the stratification of HNSCC patients and in the development of personalized treatments. We systematically analyzed the expression status of glycolysis-related genes and combined these data with clinical information, to verify the effect of the above genes on the prognosis of HNSCC.

## 2. Materials and Methods

### 2.1. Patients and Datasets

The mRNA expression profiles and clinical data from The Cancer Genome Atlas (TCGA) database (https://cancergenome.nih.gov/) are available to download. The clinical information of 528 patients is shown in Supplementary Table 1. These data were retrieved from the publicly available TCGA database; therefore, all informed consents were available.

### 2.2. Gene Set Enrichment Analysis

GSEA (http://www.broadinstitute.org/gsea/index.jsp) uses statistical methods to determine whether the genes in the gene set are enriched in the expression matrix, so as to speculate on the enrichment of the gene set in a certain biological state, and can also compare the expression difference of the gene set between groups [18]. We analyzed whether 44 nontumor tissues and 502 tumor tissues in the HNSCC cohort of the TCGA database are different in the identified gene sets. Normalized *P* value (*P* < 0.05) and normalized enrichment score (NES) were used as evaluation criteria.

### 2.3. Statistical Analysis

The expression profiles of 546 mRNAs and clinical information for 528 HNSCCs were retrieved as raw data for further analysis. We used the “caret” package in R language to divide the HNSCC samples into a training cohort and a test cohort in a ratio of 1 : 1. The expression matrices were combined with survival data. Then, univariate Cox regression analysis was performed on the training set with *P* < 0.05 to identify genes evidently related to patient survival. Next, the candidate genes were analyzed by stepwise multivariate Cox proportional regression to establish the best risk prediction model. The risk score of each sample was calculated as follows:

(1) $$\begin{array}{c} \text{risk score }=\text{ coefgene }1\times \text{expression level of gene }1+\text{coefgene }2\times \text{expression level of gene }2+\text{coefgene }3\times \\ \text{expression level of gene }3+\cdots +\text{coefgene }n\times \text{expression level of gene }\mathrm{n,} \end{array}$$

where coefgene represents the regression coefficient.

An individual risk score was calculated for each sample in the training cohort. The median risk score was used as the cut-off for defining a high-risk and a low-risk group. Kaplan–Meier analysis was conducted to compare the survival difference in the two groups. The area under the receiver operating characteristic (roc) was used to evaluate the specificity and sensitivity of the model. Univariate Cox regression and multivariable Cox regression analyses were used to examine whether the risk score was an independent prognostic factor. Subsequently, the test cohort was used to verify the accuracy of the model. Finally, stratified analysis was applied to evaluate the suitability of the risk scores for prediction of patient outcomes. In addition, changes in the identified genes in each sample were analyzed using the cBioPortal database (http://www.cbioportal.org/). All analyses were carried out based on the R software (Version 3.6.3). *P* < 0.05 was set as the threshold for statistical significance.

## 3. Results

### 3.1. Initial Screening of Genes Using GSEA

**Figure 1:**
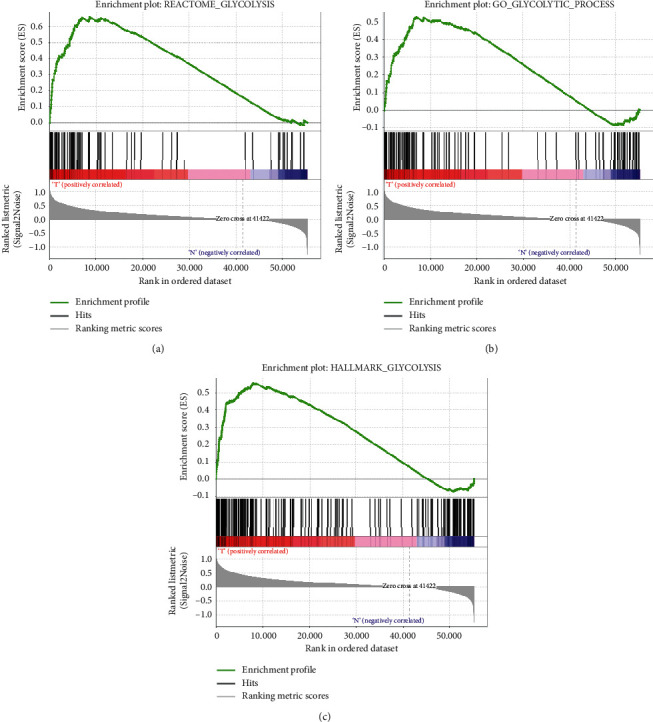
Gene set enrichment analysis (GSEA). Significant alterations in glycolysis-related gene expression were observed in head and neck squamous cell carcinoma as compared to normal tissue.

To detect differences in glycolytic metabolism during the progression of HNSCC, clinical data of 528 HNSCC patients and transcriptome data of 546 samples from the TCGA database, as well as five glycolysis-related gene sets from the molecular signature database (MSigDB), were used as the original data for further analysis. Based on GSEA enrichment analysis, we found that mRNA expression in 44 normal tissues and 502 tumor tissues was significantly different in 3 of the 5 glycolysis-related gene sets (Figure 1 and Table 1). This suggested that head and neck squamous cell carcinoma had a distinct glycolytic metabolism compared to corresponding normal tissue. Subsequently, 298 genes contained in the three glycolysis-related gene sets were further analyzed (Supplementary Table 2).

### 3.2. Identification of Prognostic Glycolysis-Related Genes

To identify novel biomarkers for outcome prediction in patients with HNSCC, 492 HNSCC patients were randomly assigned to the training and test groups at a ratio of nearly 1 : 1 (Table 2), and 298 glycolysis-related gene expression matrices were combined with survival data from the two groups. Transcriptional information and survival information for the training and test cohort are shown in Supplementary Table 3 and Supplementary Table 4, respectively. A subsequent univariate Cox regression analysis showed that 24 genes were associated with the prognosis of HNSCC patients in the training cohort (Supplementary Table 5). These prognostic glycolysis-related genes were then included in a stepwise multivariate Cox proportional regression analysis. Finally, a total of 8 genes were found to be significantly correlated with the prognosis of HNSCC and were involved in subsequent model construction. Among them, *KIF2A* and *JMJD8* were the protective genes (HR < 1), and *HMMR*, *STC2*, *HK1*, *EXT2*, *GPR8*, and *STC1* were the risk genes (HR > 1).

**Figure 2:**
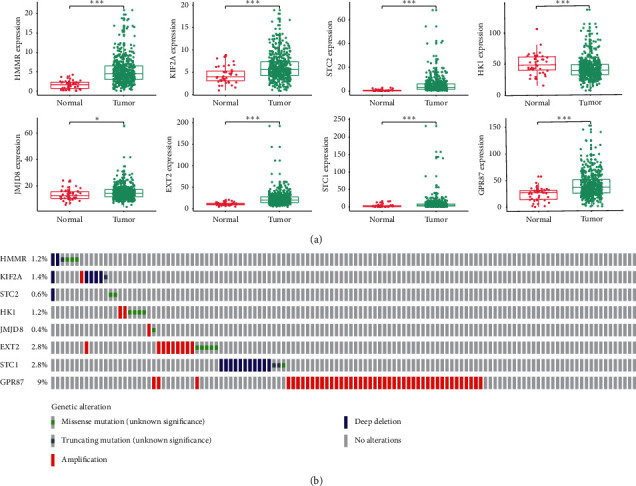
Mutations and differential expression of the 8 marker genes in normal and HNSCC tissues were investigated. (a) All eight genes showed significant differences between tumor and normal tissues. ^*∗∗∗*^, ^*∗∗*^, ^*∗*^, and ns represent *P* < 0.001, *P* < 0.01, *P* < 0.05, and *P* > 0.05, respectively. (b) *GPR87* was the most frequently mutated gene.

**Figure 3:**
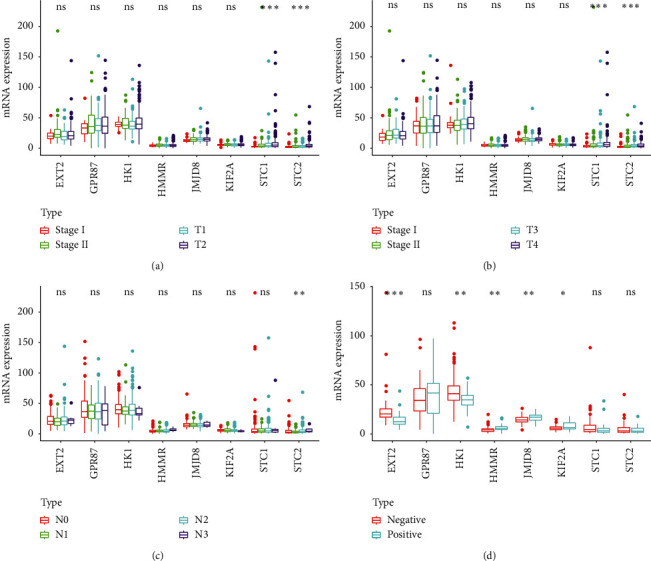
Correlation of the eight genes with different clinical characteristics: (a) clinical stage; (b) tumor size; (c) lymph node metastasis; (d) HPV status by p16 testing. ^*∗∗∗*^, ^*∗∗*^, ^*∗*^, and ns represent *P* < 0.001, *P* < 0.01, *P* < 0.05, and *P* > 0.05, respectively.

Given the potential clinical implications of the eight gene markers, their expression was compared in normal and HNSCC tissues. Except the expression of *HK1* was downregulated, the other seven genes were significantly upregulated in tumor tissues (*P* < 0.05, Figure 2(a)). Subsequently, changes in the eight selected genes were analyzed in HNSCC samples based on the cBioPortal database, revealing that amplification and deep deletion were the most common of all mutation types (Figure 2(b)). *GPR87* exhibited a mutation rate greater than 5% (9%). Finally, the eight genes were examined by clinical correlation analysis. The expression of *STC1* and *STC2* was found to significantly increase with the clinical stage (Figure 3(a)). A high expression of *STC2* was associated with larger tumors (Figure 3(b)) and the presence of lymph node metastases (Figure 3(c)). Notably, the expression of *EXT2*, *HK1*, *JMJD8*, *KIF2A*, and *HMMR* was significantly correlated with the HPV status (Figure 3(d)).

### 3.3. Generation of the Prognosis Prediction Model

The expression values of the eight genes identified above were combined with the multivariate Cox regression coefficient to obtain the risk score of each patient:

(2) $$\begin{array}{c} \text{risk score}=\text{0.}096146079\times \text{expression level of HMMR}+\left(-\text{0.}134494599\right)\times \text{expression level of KIF}2\text{A}+\text{0.}034033864\times \text{expression level of STC}2\;+\text{ 0.}008524517\times \text{expression level of HK}1+\left(-\text{0.}047903542\right)\times \text{expression level of JMJD}8+\text{0.}011711524\times \text{expression level of EXT}2+\text{0.}026067236\times \text{expression level of STC}1+\text{0.}007433349\times \text{expression level of GPR}87\text{.} \end{array}$$

**Figure 4:**
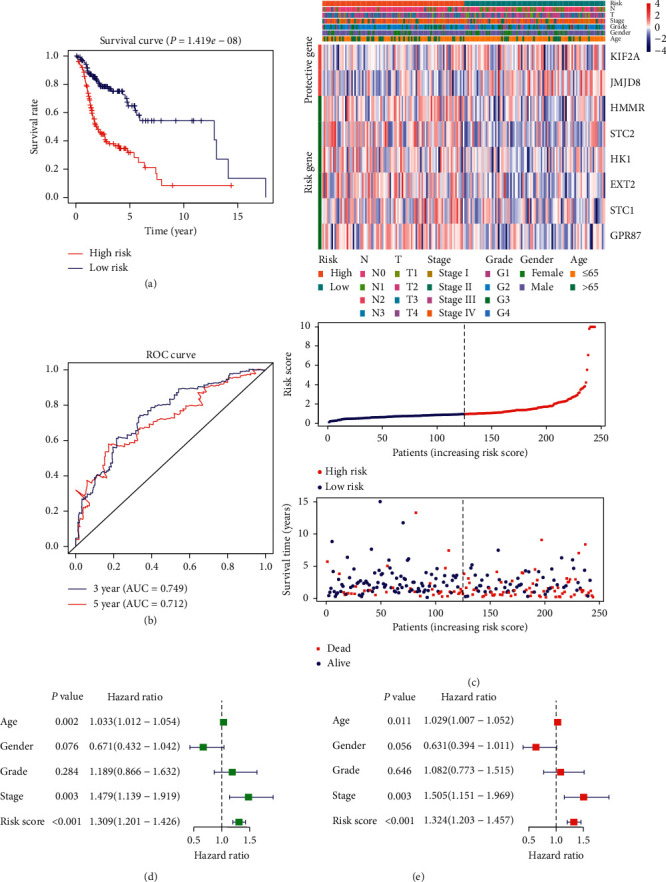
Prognosis model was constructed from HNSCC sample data from the training cohort: (a) analysis of prognostic differences after classification based on the training set; (b) the results of ROC curve prove the accuracy of the model; (c) the expression of 8 genes and the survival of patients varied with the change in risk score; (d) forest plot of univariate Cox regression analysis in the training cohort; (e) forest plot of multivariate Cox regression analysis in the training cohort.

Using the median risk score as the cut-off point, HNSCC patients in the training cohort were divided into high-risk and low-risk groups. Subsequent Kaplan–Meier analyses showed that overall survival was significantly lower in the high-risk group than in the low-risk group (*P* < 0.001, Figure 4(a)). For each sample, the results of ROC analysis showed that the prognostic index based on glycolytic-related genes was a potential survival predictor, with an AUC of 0.749 and 0.712 for 3- and 5-year survival, respectively (Figure 4(b)). The samples were then sequenced from low to high based on the risk score to identify whether the gene expression level and patient's survival varied with the risk score (Figure 4(c)). With an increase in risk score, the expression levels of *KIF2A* and *JMJD8* decreased, whereas those of *HMMR*, *STC2*, *HK1*, *EXT2*, *GPR8*, and *STC1* increased. Moreover, the number of patient deaths increased with the risk score.

To compare the ability of risk scores and conventional clinical indicators to predict the outcome of patients with HNSCC, univariate and multivariate Cox hazard analyses were used to examine the value of these indicators. Univariate analysis showed that age, clinical stage, and risk score were effective prognostic indicators (Figure 4(d)). In the multivariate analysis after correcting the clinical characteristics included in the analysis, age, clinical stage, and risk score still had significant prognostic significance and could be used as independent prognostic indicators, among which the risk score had the best prognostic ability (Figure 4(e)).

### 3.4. Evaluation of the Prognosis Prediction Model

**Figure 5:**
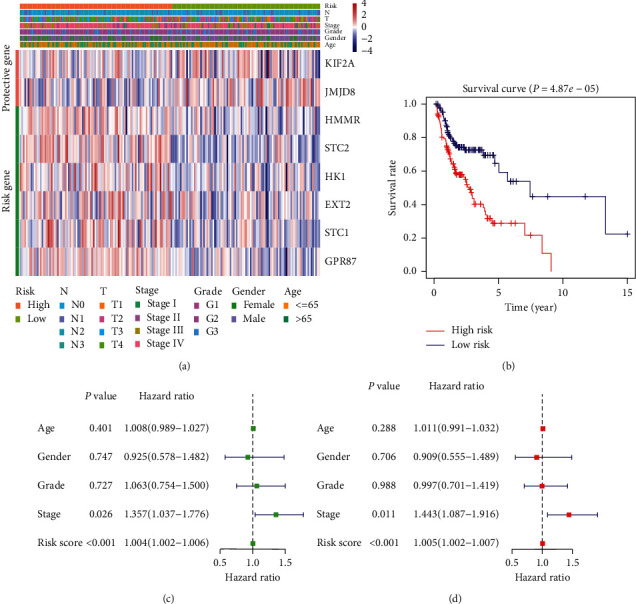
HNSCC samples from the test cohort demonstrated the stability of the prognostic model: (a) analysis of the prognostic differences after classification in the testing cohort; (b) the expression levels of 8 genes in the test cohort varied with the risk score; (c) forest plot of univariate Cox regression analysis in the test cohort; (d) forest plot of multivariate Cox regression analysis in the test cohort.

First, risk scores were calculated using the expression of eight selected genes in the test cohort and prognosis models based on the training cohort. Then, the samples were sequenced from low to high according to the risk score, and the expression pattern of 8 genes is shown in Figure 5(a). Similarly, the samples were divided into the high- and low-risk groups with the median risk score as the cut-off point. Again, Kaplan–Meier analysis showed that the overall survival of the two groups was also significantly different (Figure 5(b), *P* < 0.001). Finally, we observed that both with univariate (Figure 5(c)) and multivariate Cox hazard analyses (Figure 5(d)), risk scores based on eight genes did have significant prognostic power. These results are in accordance with the results of the training cohort, which proves that the model based on 8 genes is stable and reliable.

### 3.5. Role of Survival Prediction Based on the Risk Score of the 8-mRNA Signature

**Figure 6:**
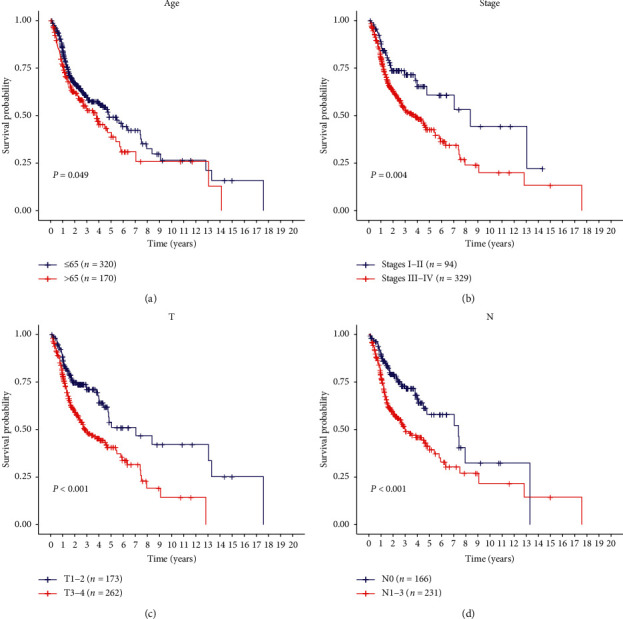
Kaplan–Meier survival analysis based on several different clinical characteristics of all HNSCC patients. Age (a), clinical stage (b), tumor size (c), and lymph node metastasis (d) can predict patients' survival to some extent.

**Figure 7:**
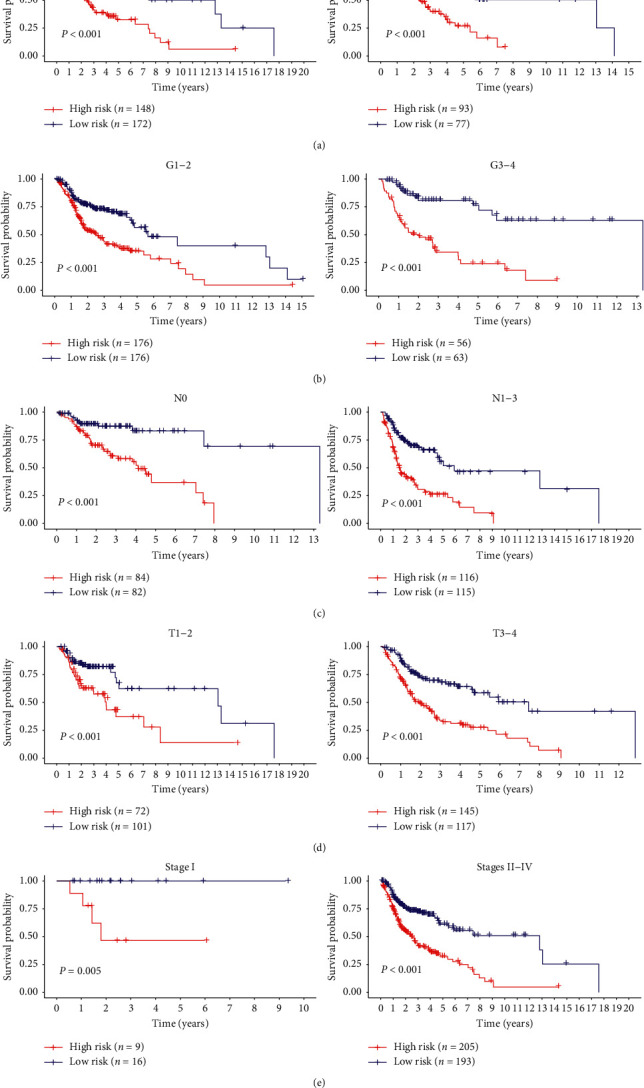
Predictive power of risk scores after stratification of various clinical features: (a) age; (b) histologic grade; (c) lymph node metastasis; (d) tumor size; (e) clinical stage.

The Kaplan–Meier curves revealed that age (≥65, *P*=0.049), clinical stage (III and IV, *P*=0.004), tumor size (T3 and T4, *P* < 0.001), and lymph node metastasis (N, *P* < 0.001) were correlated with poor prognosis (Figures 6(a)6(d)). Notably, the risk score based on the 8-gene signature was better at predicting the survival of HNSCC patients, as compared to the above clinical factors (Figures 4(a) and 5(b)). Subsequently, we performed a hierarchical analysis of patients to validate the reliability of the risk score in predicting HNSCC prognosis. As shown by the K-M curve, when the patients were stratified based on age, gender, histologic grade, and clinical stage, the risk score retained stable prediction power among HNSCC patients in various states (Figures 7(a)7(e)). Crucially, the risk score also had an excellent prognostic power on the early stage of HNSCC (Figure 7(e)).

## 4. Discussion

The metabolic switch from oxidative phosphorylation (OXPHOS) to aerobic glycolysis is an emerging hallmark of cancer cells [19]. Although the amount of ATP generated by glycolysis is low, several advantages inherent in aerobic glycolysis may explain this metabolic switch in cancer cells. First, glycolysis produces ATP 100 times faster than OXPHOS [20] and could provide sufficient energy for cell survival. Second, glycolytic intermediates could be transferred to various biosynthetic pathways, providing material for the synthesis of biological macromolecules and organelles [21, 22]. Moreover, the intermediates that cancer cells accumulate during glycolysis promote the pentose phosphate pathway, ensuring that it grows in an environment with adequate reduced glutathione levels. The latter molecule plays a key role in protecting cancer cells from oxidative damage and antitumor drugs [23, 24]. Finally, the formation of an acidic microenvironment associated with lactic acid accumulation owing to increased glycolysis provides a tissue environment for tumor recurrence and tumor metastasis potential [25, 26]. These factors increase the dependence of tumor cells on glycolysis and provide a biochemical basis for prioritizing the killing of cancer cells by using glycolysis as a therapeutic target, which potentially improves the therapeutic effects.

HNSCC is a refractory tumor, one of the deadliest malignancies in humans, and its overall survival rate is extremely low. This is due to the high incidence of local recurrence and distant metastasis [27]. In order to improve HNSCC treatment, reliable clinical biomarkers are urgently needed, which will be helpful for the clinical diagnosis, prognosis, assessment of relapse prediction, and clinical intervention. Glycolysis is closely related to HNSCC [28]. Although majority of researchers have concentrated on the molecular mechanisms of glycolysis in tumorigenesis, proliferation, and invasion, evidence for a potential association between glycolysis and the survival of HNSCC patients has also been reported. For example, high expression levels of *TKTL1*, *GLUT*-1, *MCT1*, and *MCT4* are associated with unfavorable prognosis in HNSCC patients [17, 29–31]. Given the importance of glycolysis in HNSCC, it is reasonable to speculate that glycolysis-associated genes hold great promises as predictors of HNSCC outcomes. Moreover, multiple-gene signatures derived from reliable algorithms are superior to single molecules in predicting overall survival [32]. In this study, the mRNA expression profiles of 298 glycolysis-associated genes were analyzed in a TCGA head and neck squamous cell carcinoma cohort. Eight genes related to glycolysis were selected as candidate prognostic predictors in HNSCC. These genes are potential molecular biomarkers of prognosis and therapeutic targets and may help develop individualized treatments based on patient risk.

For most of the eight glycolysis-related genes identified herein, a prognostic role in HNSCC or other malignancies has been previously reported. GPR87 is a cell surface G protein-coupled receptor that is highly expressed in a variety of tumors and plays a crucial role in the survival of tumor cells [33]. Nii et al. reported that the overexpression of GPR87 in non-small cell lung carcinoma is significantly correlated with poor patient survival [34]. Hyaluronan-mediated motility receptor (HMMR) is a regulator of homeostasis, mitosis, and meiosis, and its dysfunction may promote tumorigenesis and cancer progression [35]. The expression of *HMMR* may be an effective prognostic marker in progression-free survival of patients with the papillary subtype of bladder cancer [36]. Hexokinase (HK), a rate-limiting enzyme catalyzing the first step of glycolysis, has four known subtypes: HK1–HK4 [37]. Although most studies focused on HK2, some studies found that the expression of *HK1* is connected with disease progression, invasion, and poor survival in patients with esophageal squamous cell carcinoma [38]. *JMJD8,* a member of the Jumonji C domain-containing (JMJD) protein family, regulates glycolysis metabolism by interacting with pyruvate kinase M2 and becomes upregulated during *in vitro* endothelial cell differentiation and stimulates angiogenic sprouting [39]. Similarly, *JMJD8* downregulation reduces the viability of DU145 prostate cancer cells [40]. Exostosin (EXT) proteins are glycosyltransferases, which regulate intracellular signaling, cell-cell interactions, and tissue morphogenesis [41]. Mutations in exostosin-2 (EXT2) often cause multiple osteochondromas [42, 42]. At the same time, Huang et al. showed that EXT2 is an independent prognostic factor for hepatocellular carcinoma [43, 43]. Stanniocalcin (STC) is a glycosylated peptide hormone involved in calcium and phosphate homeostasis [44]. Among them, STC2 can regulate glucose homeostasis [45]. Studies have found that high expression of *STC2* is associated with tumor invasion, metastasis, and poor prognosis [46]. STC1 uncouples the process of oxidative phosphorylation via an increased expression of mitochondrial UCP2 [47]. Su et al. reported that STC1 is a valuable biomarker for the diagnosis of malignant glioma and the evaluation of prognosis after surgery [48]. In Table 3, we briefly summarized the eight glycolysis-related genes.

The emphasis of this study was to investigate the role of the expression of glycolysis-related genes in the prognosis of HNSCC. The mRNA expression of eight genes was significantly different between tumor and normal tissues, and these changes were consistent with a role of glycolysis in the development of HNSCC. Genetic changes may affect mRNA expression, and gene amplification is usually correlated with mRNA upregulation, in line with our results. In addition, our clinical correlation study revealed that the expression of certain genes was significantly correlated with specific clinical characteristics, especially those related to HPV, which may inspire future studies focusing on the role of glycolytic genes in cancer.

However, our research has certain limitations: first, the prognostic model we developed needs to be validated in additional independent samples; second, the pathogenic role of the identified glycolysis-related genes was not characterized at the molecular level; third, our research was a retrospective study and may contain inherent biases; fourth, the prediction model reported in this study needs to be improved in actual clinical tests.

In conclusion, we have identified eight prognostic genes and constructed a new risk scoring model for HNSCC patients based on a series of bioinformatics analyses, correlating the expression profiles of glycolysis-associated genes with various clinical features. Our study was the first to demonstrate that glycolysis-related transcriptional patterns may affect the prognosis of patients with HNSCC. The identified genes may also inspire the development of new therapeutic approaches for HNSCC. In conclusion, our findings may help improve prognosis and diagnosis, as well as develop personalized therapies for patients with HNSCC.

## Figures and Tables

**Table 1:** Enrichment results of 5 glycolysis-related gene sets.

Name	Size	ES	NES	NOM *P*-val	FDR *q*-val	FWER *P*-val
HALLMARK_GLYCOLYSIS	200	0.5581	2.0104	0	0	0
REACTOME_GLYCOLYSIS	72	0.6599	1.9791	0	0	0
GO_GLYCOLYTIC_PROCESS	106	0.5277	1.6752	0.0161	0.0161	0.01
KEGG_GLYCOLYSIS_GLUCONEOGENESIS	62	0.3501	1.1905	0.2145	0.2145	0.071
BIOCARTA_GLYCOLYSIS_PATHWAY	3	0.5512	0.8949	0.6477	0.6477	0.342

ES, enrichment score; NES, normalized enrichment score; NOM *P*-val, nominal *P*-value; FDR *q*-val, false discovery rate q-value; FWER *P*-value, family-wise error rate *P*-value.

**Table 2:** Comparison of clinical characteristics between the training cohort and the test cohort.

		Training cohort	Text cohort
Cases		248	244
Age	≤65	168	155
	>65	80	90
Gender	Female	63	67
	Male	185	177
Grade	G1	32	28
	G2	141	153
	G3	62	55
	G4	2	0
Stage	Stage I	15	10
	Stage II	33	36
	Stage III	42	36
	Stage IV	124	128
T (tumor)	T1	25	19
	T2	61	69
	T3	49	47
	T4	86	80
N (lymph node)	N0	79	88
	N1	38	27
	N2	78	82
	N3	5	2
HPV status by p16 testing	Negative	42	30
	Positive	12	18

**Table 3:** Introduction and summary of eight genes constructing the prognostic model.

Gene symbol	Full name	Encoding protein	Function	Risk coefficient
GPR87	G protein-coupled receptor 87	A cell surface G protein-coupled receptor	Glycolysis-related proteins	0.007433349
HMMR	Hyaluronan-mediated motility receptor	Hyaluronan-mediated motility receptor	Regulate homeostasis, mitosis, and meiosis	0.096146079
HK1	Hexokinase-1	A member of the hexokinases	The first rate-limiting enzyme in glycolysis	0.008524517
JMJD8	Jumonji C domain-containing 8	A member of the Jumonji C domain-containing (JMJD) protein family	Regulating glycolysis metabolism by interacting with pyruvate kinase M2	−0.047903542
EXT2	Exostosin-2	A member of the exostosin family	An enzyme that harbors glycosyltransferase activities	0.011711524
STC1	Stanniocalcin 1	A glycosylated peptide hormone	Uncouples the process of oxidative phosphorylation	0.026067236
STC2	Stanniocalcin 2	A glycosylated peptide hormone	Regulating glucose homeostasis	0.034033864
KIF2A	Kinesin family protein 2A	A member of the kinesin-13 family	Glycolysis-related proteins	−0.134494599

## Data Availability

This study used public data accessible in The Cancer Genome Atlas (TCGA) database.

## Supplementary Materials

Supplementary Materials Supplementary Table 1: clinical information for 528 samples.xlsx. Supplementary Table 2: expression of 298 glycolysis-related genes in 546 HNSCC samples.xlsx. Supplementary Table 3: the expression of glycolysis-related genes and survival of patients in the training cohort.xlsx. Supplementary Table 4: the expression of glycolysis-related genes and survival of patients in the text cohort.xlsx. Supplementary Table 5: univariate Cox regression analysis of the training dataset (24 mRNAs were identified, P0.05).xlsx.
